# Supplementary material for: Nurse-Led Bereavement Support During the Time of Hospital Visiting Restrictions Imposed by the COVID-19 Pandemic—A Qualitative Study of Family Members’ Experiences
Source: Nurs Rep. 2025 Jul 14;15(7):254. doi: 10.3390/nursrep15070254 (PMC12299844; doi:10.3390/nursrep15070254)
Supplement: Supplementary file 1 [file nursrep-15-00254-s001.zip › nursrep-3693204-supplementary.pdf]

To the Kind Attention
Of the Family of
[redacted]

Dear Family,

We would like to extend our heartfelt condolences on behalf of the Istituto Cardiocentro Ticino and, in particular, the entire care team.

We are aware of the difficulties and discomforts that a situation of mourning can cause, and we would like to give a concrete sign of our sharing and support while respecting discretion and privacy.
The current restriction of social contacts, and therefore of visits to hospitals, imposed by the management of the pandemic, has certainly added further frustration to the experience of you family members at home.
Therefore, in the coming days, you will be contacted by the nursing staff who will be available to listen to you and provide any clarifications regarding [redacted]'s hospitalization at our facility.

We reiterate your total freedom in accepting this invitation and any unavailability will still be understood by us.

We would like to express our condolences and extend our best regards.

Head Nurse
[redacted]

Lugano, 30.03.2020

For communications and information, please write to the following addresses:
[redacted]
